# Dichotomous Effect of Caffeine, Curcumin, and Naringenin on Genomic DNA of Normal and Diabetic Subjects

**DOI:** 10.1155/2014/649261

**Published:** 2014-03-30

**Authors:** Debarati Chattopadhyay, Ashok Somaiah, Divya Raghunathan, Kavitha Thirumurugan

**Affiliations:** Structural Biology Lab, Centre for Biomedical Research, School of Bio Sciences & Technology, VIT University, Vellore, Tamil Nadu 632014, India

## Abstract

Nutraceutical compounds show antioxidant and prooxidant properties under stress conditions like cancer, diabetes, and other diseases. The objective of this study is to find the dichotomic behavior of caffeine, curcumin, and naringenin on DNA of diabetic and normal subjects in the presence and absence of copper, hydrogen peroxide, and complex of copper-hydrogen peroxide. Hydrogen peroxide releases hydroxyl free radicals (^•^OH) on oxidation of Cu (I) to Cu (II) through Fenton-type reaction to cause DNA damage. In the results, agarose gel electrophoretic pattern speculates the prooxidant effect of caffeine and antioxidant effect of curcumin on DNA in the presence of copper and hydrogen peroxide. UV-Vis spectral analysis shows hyperchromism on addition of DNA to caffeine, hypochromism with curcumin, and subtle changes with naringenin. The chosen nutraceuticals act as inducers and quenchers of oxidative free radicals arising from diabetes.

## 1. Introduction

Modern sedentary life-style coupled to high calorie diet aggravates oxidative stress level culminating in avoidable diseases like obesity, diabetes, hypertension, cataract, and cancer. The oxidative stress caused by diabetes is accompanied by production of reactive oxygen species (ROS) (hydroxyls, superoxides, peroxides), which can generate potential damage to mammalian deoxyribonucleic acid (DNA) [1]. Oxidative stress arises due to an imbalance between production and consumption of ROS and presence of metals exacerbates the generation of free radicals. Copper is one of the most redox-active metal ions present in cells and closely associated with chromatin. Copper concentration in tissues increases in various malignancies [2]. Hydroxyl radicals (^∙^OH) are released in the superoxide driven Fenton reaction, where Cu (I) gets oxidized to Cu (II) in the presence of hydrogen peroxide [3] and causes DNA damage. Because copper ions catalyze hydroxyl radical formation,* in vivo* the copper level is maintained low to protect the cell. Hydrogen peroxide has poor radical activity, but at higher concentration (>50 *μ*M) through the formation of hydroxyl radical, it can cause DNA strand breakage and base modification [4]. The extent of hydroxyl radicals causing damage to DNA of diabetic patients is higher compared to healthy humans [5]. ROS is involved in blocking the pathway between insulin-receptor substrate (IRS-1) and phosphatidylinositol-4,5-bisphosphate 3-kinase to result in high insulin resistance [6]. Antioxidant enzymes (superoxide dismutase, glutathione peroxidase, catalase) and nonenzymatic antioxidants (vitamin E, vitamin C, glutathione) offer detoxification to a certain extent. When excess free radicals are produced, these antioxidant systems are not adequate to meet the requirement, necessitating consumption of natural food source rich in polyphenols. Polyphenol compounds are routinely studied for their radical scavenging activity [7]. There is a growing interest to find the mode of interaction between phytochemical compounds and DNA. In our study, the hypothesis was proposed to know the potential of caffeine, curcumin, and naringenin to protect the DNA damage that may arise from stress related to diabetes.

Coffee is the most happily consumed beverage worldwide. Hence, knowing the beneficial and harmful effect of its intake on human health is crucial. Caffeine (1,3,7-trimethylxanthine) is a bitter, white crystalline alkaloid present as a legal stimulant in coffee. Caffeine contributes to the antioxidant value of coffee and removal of caffeine resulted in 25–30% reduction in antioxidant value [8]. Regular intake of coffee (mean daily intake ~480 mL) contributes to 64% of total antioxidant capacity in humans [9]. Presence of caffeine reduces insulin sensitivity in humans [10, 11]. In contrast, there are reports on the beneficial effect of caffeine consumption on type 2 diabetic patients, where it lowers the diabetes risk [12–15]. Caffeine ingestion reduced whole-body glucose disposal to 24% and carbohydrate storage by 35%, compared to placebo administration [16]. There is a marked reduction in blood glucose level of diabetic patients consuming caffeinated instant coffee for 16 weeks [17].

Curcumin (diferuloylmethane) is a natural polyphenol compound present in turmeric. Presence of ortho-methoxy phenolic OH and *β*-diketone moiety of curcumin offers the ability to directly quench ROS and prevent oxidative damage [18]. Curcumin can exist in many tautomeric forms; the most commonly occurring forms are the 1,3-di-keto form and two enol forms. The di-keto form is primarily involved in deprotonation and enolates, thereby contributing to its active antioxidative capabilities. The ability of curcumin to reduce blood sugar level dates back to early years [19] and curcuminoids (NCB-02) intake (150 mg/twice a day) by diabetic patients improved endothelial function and reduced the oxidative stress and inflammatory markers [20]. Curcumin binds with DNA along the “minor groove” and it may cause DNA damage in the cell at high concentration [21].

Naringenin (4′,5,7-trihydroxyflavanone) is a flavanone present in many citrus fruits, for example, grapefruit [22]. Peak plasma concentration for naringenin following ingestion of grapefruit juice indicates its beneficial effect [23]. Naringenin improved glucose stimulated insulin secretion and glucose sensitivity in INS-1E cells [24]. Naringenin shows anti-inflammatory effect in STZ-induced diabetic rats by blocking NF-*κ*B pathway [25].

In our study, we have investigated the effect of three nutraceutical compounds (caffeine, curcumin, and naringenin) on DNA in the presence and absence of copper sulphate through agarose gel electrophoresis and UV-visible spectrophotometry.

## 2. Materials and Methods

### 2.1. Isolation of Genomic DNA

Genomic DNA was isolated from whole blood sample of diabetic patients and healthy individuals using phenol/chloroform method. The research followed the ethical standards formulated in the Helsinki Declaration of 1964, revised in 2000, and written consent was obtained from the human subjects before collecting the blood samples. The clinical characteristics of the studied subjects were shown in Table 1. Though the number of subjects studied is limited, volume of blood collected was enough to isolate required genomic DNA. Each vial containing DNA was dissolved in 1X TBE buffer and stored in −20°C for further use. The purity of DNA measured using UV-visible spectrophotometer was in the range of 1.9-2.0.

### 2.2. Stock Solutions

Caffeine (1,3,7-trimethylxanthine) (HiMedia: RM6753), curcumin (diferuloylmethane) (Sigma: C-1386), and naringenin (4′,5,7-trihydroxyflavanone) (Sigma: N-5893) were all diluted to 10 mM stock solutions with deionized water just prior to use. To avoid the radical scavenging activity of solvents such as DMSO or ethanol, addition of a small amount of NaOH (20–100 *μ*L of 1 M NaOH per 10 mL polyphenol stock solution) was sufficient to quickly and completely dissolve curcumin and naringenin. Stock solution of hydrogen peroxide [10 mM] was prepared fresh in PBS solution [10 mM] and copper sulphate [10 mM] was dissolved in water before use. The phytochemicals were stored in 4°C, and wrapped with aluminum foil to reduce their sensitivity to light.

### 2.3. Induction of DNA Damage

The reaction mixture (20 *μ*L) for agarose gel electrophoresis consists of genomic DNA (15 *μ*g), H_2_O_2,_ phytochemical compounds (caffeine, curcumin, and naringenin), and CuSO_4_ at equimolar concentrations (0.05 mM–6 mM) in phosphate buffered saline at pH 7.4. Incubation was carried out at 37°C for 30 minutes followed by exposure to ultraviolet radiation for 30 minutes to ensure maximum damage. After incubation, samples were loaded onto agarose gel electrophoresis (1.2%). Untreated genomic DNA served as a control. Each sample was run three times to ensure reproducibility and reliability. The gels were placed in transilluminator to observe DNA damage.

### 2.4. UV-Vis Absorbance Studies

UV-visible spectra of the interaction between phytocompounds (final concentration: 50 *μ*M) and genomic DNA at various final concentrations (18 *μ*g, 36 *μ*g, 72 *μ*g) in PBS buffer (700 *μ*L) were measured after incubation for 1 h at 37°C. The interaction between these phytochemical compounds with normal and diabetic genomic DNA in the presence of CuSO_4_ (50 *μ*M) was also detected after 1 h incubation at 37°C. DNA was allowed to interact with phytocompounds for 5 minutes to ensure complex formation, before it was exposed to copper sulphate. Spectral reading was performed using Evolution 260 Bio Thermo Scientific spectrophotometer in the scan range wavelength of 200–500 nm.

## 3. Results 

### 3.1. Agarose Gel Electrophoresis

**Figure 1:**
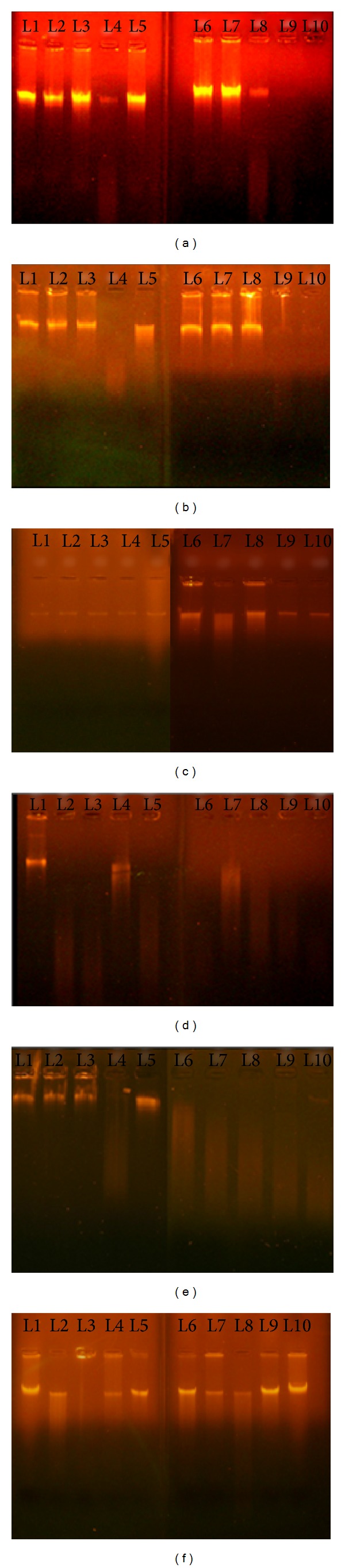
Agarose gel electrophoresis (1.2%) of genomic DNA from nondiabetic human ((a), (b), (c)) and diabetic subject ((d), (e), (f)). L1: Genomic DNA (untreated, control, 15 *μ*g). L2: DNA (15 *μ*g) + H_2_O_2_ (4 mM). L3: DNA (15 *μ*g) + CuSO_4_ (4 mM). L4: DNA (15 *μ*g) + H_2_O_2_ (4 mM) + CuSO_4_ (4 mM). L5: DNA (15 *μ*g) + H_2_O_2_ (50 *μ*M) + *Phytocompound (50 *μ*M) + CuSO_4_ (50 *μ*M). L6: DNA (15 *μ*g) + H_2_O_2_ (500 *μ*M) + *Phytocompound (500 *μ*M) + CuSO_4_ (500 *μ*M). L7: DNA (15 *μ*g) + H_2_O_2_ (1 mM) + *Phytocompound (1 mM) + CuSO_4_ (1 mM). L8: DNA (15 *μ*g) + H_2_O_2_ (2 mM) + *Phytocompound (2 mM) + CuSO_4_ (2 mM). L9: DNA (15 *μ*g) + H_2_O_2_ (4 mM) + *Phytocompound (4 mM) + CuSO_4_ (4 mM). L10: DNA (15 *μ*g) + H_2_O_2_ (6 mM) + *Phytocompound (6 mM) + CuSO_4_ (6 mM). *Caffeine, curcumin, and naringenin.

It has been known that redox metals like iron, zinc, and copper play a major role in exacerbating the DNA damage along with hydrogen peroxide by Haber-Weiss and Fenton reaction [26]. Polyphenols have been found to mobilize endogenous copper and bring about prooxidant activity on reduction of Cu (II) to Cu (I). Three polyphenol compounds (caffeine, curcumin, and naringenin) were evaluated for their potential to cause or prevent damage on normal and diabetic genomic DNA. In the case of nondiabetic DNA (Figures 1(a), 1(b), and 1(c)), hydrogen peroxide or copper sulphate alone cannot cause the damage (L2, L3), but they can cause marked damage (shearing) when used in combination (L4). At low concentration of caffeine, curcumin (50 *μ*M) with equimolar hydrogen peroxide, and copper sulphate, the damage was not observed (Figures 1(a) and 1(b); L5), but they displayed shearing at higher concentrations (4 mM, 6 mM) (Figures 1(a) and 1(b); L9, L10). Caffeine showed the damage even at 2 mM concentration (Figure 1(a), L8). Surprisingly, naringenin offers protection at higher concentrations (Figure 1(c), L9, L10), but it causes damage from 500 *μ*M to 2 mM (Figure 1(c), L6–L8). In the DNA sample obtained from diabetic patients (Figures 1(d), 1(e), and 1(f)), adding either hydrogen peroxide or copper sulphate resulted in shearing of DNA (Figures 1(d) and 1(f); L2, L3). In combination, they had displayed the damage (Figures 1(d) and 1(e); L4), suggesting the significant role played by copper in mediating havoc. There is a pronounced shearing effect when caffeine, curcumin, and naringenin used equimolar along with hydrogen peroxide and copper sulphate at concentration from 500 *μ*M to 6 mM (Figures 1(d), 1(e), and 1(f); L6–L10). Caffeine starts to show damage even at low concentration of 50 *μ*M (Figure 1(d), L5). This is in contrast to the observed behaviour in nondiabetic DNA.

### 3.2. UV-Vis Absorbance Studies

**Figure 2:**
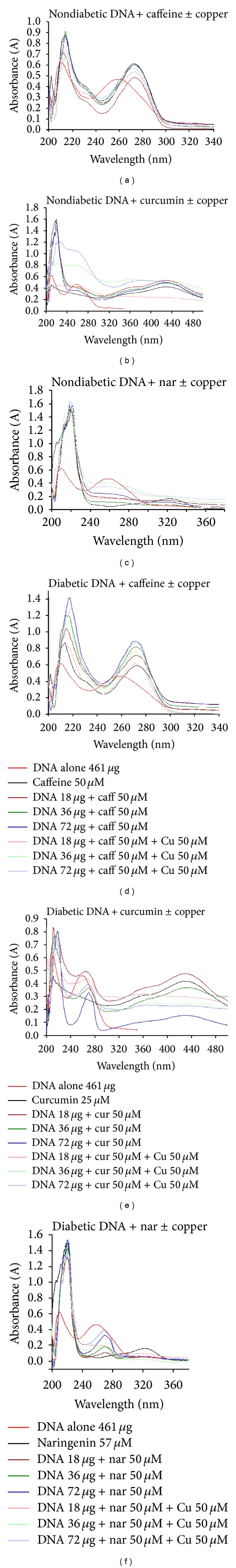
Spectra of caffeine, curcumin, and naringenin with DNA from nondiabetic ((a), (b), (c)) and diabetic subject ((d), (e), (f)) in the presence and absence of copper.

Caffeine exhibited an absorption maximum at 272 nm. On addition of DNA sample at varying concentrations to caffeine, the absorbance increased linearly in proportion to increase in DNA concentration (Figures 2(a) and 2(d)). The same pattern was observed in the presence of copper. Curcumin displayed an absorption maximum at 430 nm. When DNA was added to curcumin, the spectral absorbance reduced linearly with increasing DNA concentration (Figures 2(b) and 2(e)). This is in contrast to the absorbance pattern of caffeine. On addition of copper to DNA-curcumin, the peak disappeared. Naringenin showed an absorption maximum at 321 nm. On addition of diabetic DNA sample at varying concentrations to naringenin, the absorbance increased linearly with increase in DNA concentration and new peak emerged at 270 nm both in the presence and absence of copper (Figure 2(f)). The characteristic peak of naringenin alone at 321 nm was not observed in DNA-naringenin with and without copper (Figures 2(c) and 2(f)).

## 4. Discussion

### 4.1. Agarose Gel Electrophoresis

When genomic DNA of nondiabetic subjects was treated with equimolar concentrations of hydrogen peroxide or copper sulphate (4 mM each), there was no damage observed (Figures 1(a), 1(b), and 1(c); L2, L3), but marked shearing was observed in the presence of hydrogen peroxide and copper sulphate together at 4 mM (Figures 1(a) and 1(b); L4). These results imply that copper ions might react with hydrogen peroxide to generate DNA-damaging hydroxyl free radicals. Use of caffeine and curcumin failed to rescue the damage at the same concentration (4 mM) but offered protection at the concentrations of 50 *μ*M, 500 *μ*M, and 1 mM. Polyphenols when used on their own may not have the strong ability to bind DNA and cause damage, but in the presence of copper, these compounds confer damage through redox-cycling pathway of copper ions in a concentration dependent manner. In the case of DNA samples obtained from diabetic subjects, hydrogen peroxide or copper sulphate on their own and in combination produced damage (Figures 1(d) and 1(f); L2–L4). This speculates that DNA of diabetic sample is much prone to be attacked readily by stress inducing chemicals. And on treatment with caffeine or naringenin at the same concentration (4 mM), these phytocompounds failed to relieve the damage (Figures 1(d) and 1(f); L9). Curcumin behaves as antioxidant at 50 *μ*M concentration (Figure 1(e), L5), whereas caffeine acts as prooxidant at the same concentration (Figure 1(d), L5), but all the three phytocompounds produced severe damage at higher concentrations starting from 500 *μ*M to 6 mM. This suggests that caffeine and curcumin confer dichotomous effect (prooxidant and antioxidant) against DNA damage induced in the presence of copper and hydrogen peroxide. The reason for observing DNA damage at lower concentrations might be due to the insufficient number of available caffeine molecules to scavenge hydroxyl radicals, so that these radicals are left free to cause damage to DNA.

Hyperglycemia induced oxidative stress promotes production of reactive oxygen species (ROS) [27]. Like Janus, the two-faced roman God, ROS gives beneficial and harmful effect when being present in low and high concentrations, respectively. At low concentrations, they help in combating infectious agents thus protecting the cell and at high concentrations they cause oxidative stress. Overproduction of ROS in the presence of limited availability of antioxidants augments the oxidative stress induced damage on proteins, lipids, and DNA, impairing their function [28]. This demands the need for maintaining “redox equilibrium” between pro-oxidant system and antioxidant system, which plays a key role in various diseases like aging, obesity, diabetes, and cancer. Prooxidant activity of polyphenols in the presence of copper has been studied [29]. According to the structure-activity relationship of compounds in polyphenols, compounds bearing hydroxyl groups are found to be most active in inducing DNA damage in the presence of copper [30]. This high prooxidant activity of polyphenols is due to the presence of hydroxyl groups, which are capable of chelating with copper to form polyphenol-copper complex that enables intramolecular electron transfer to form semiquinone radical anion. Reacting with O_2_, the radical undergoes a second electron transfer to form quinones and O^2−^. At neutral pH, O^2−^ protonates and forms H_2_O_2_. H_2_O_2_ may also be formed by dismutation of O^2−^. Thus, H_2_O_2_ can immediately participate in Fenton-type ^∙^OH formation and DNA cleavage reaction. In addition, quinones are reportedly involved in DNA damage by forming covalent adducts with DNA [31]. In brief, the formation of the hydroxyl radical and the copper-redox cycle play a key role in inducing DNA damage. Among the oxygen radicals, the hydroxyl group is the most electrophilic radical with high reactivity and possesses a small diffusion radius. Thus to cleave DNA, the hydroxyl radical should be produced in the vicinity of DNA [32]. Green tea polyphenols have the ability to cause oxidative DNA damage in the vicinity of Cu (II) [33].

### 4.2. UV-Vis Absorbance Studies

Our spectral results show the increased absorbance (hyperchromism) observed on addition of DNA to caffeine both in the presence and absence of copper (Figures 2(a) and 2(d)). We speculate this might be due to molecular aggregation and hydrogen bond damage. There is a gradual reduction in spectral absorbance (hypochromism) on binding of curcumin to DNA (Figures 2(b) and 2(e)). This implies the possibility of intercalation among the bases and *π*-π* interaction between DNA and curcumin [34]. Disappearance of peak on addition of copper to DNA-curcumin complex suggests weaker affinity between oxygen ligand of curcumin and copper ion (soft Lewis acid) [35]. Effect of copper on phytocompounds interaction with DNA is important to know the extent of metal influence on stability. It was expected that DNA being a negatively charged polyanion could attract cations, thereby favouring the strong interaction. There is a conflicting report on the function of curcumin-copper complex. Curcumin-Cu (II) complex had shown prooxidant effect [36]. Curcumin-copper complex (1 : 1 and 1 : 2) displayed* in vitro* antioxidant activity and superoxide dismutase (SOD) activity [37]. There is a marked hypochromic shift in the presence of progressively increasing concentration of DNA due to close connection of *π* electron cloud of curcumin with DNA base pairs [38]. They did not observe any unwinding of DNA and conformational changes on binding to curcumin and hydrophobic forces govern the stability of interaction and not electrostatic forces. In the interaction studies of DNA with naringenin, there is increase in absorbance (Figures 2(c) and 2(f)). This might be due to unwinding of DNA and exposure of the bases. The disappearance of peak at 321 nm and emergence of new peak at 270 nm in DNA of diabetic subject with naringenin (with and without copper) implies complex formation. Effect of various metals (Cu^2+^, Co^2+^, Ni^2+^, K^+^) on binding of naringenin to DNA did not show change in binding constant [39]. Spectral studies of Cu (II)-naringin complex and DNA displayed intercalative mode of interaction through N(7) of guanine site [39].

From the gel studies, we observe that the complex DNA-caffeine acts as prooxidant even at lower concentration (50 *μ*M), and the complex DNA-curcumin/naringenin acts as antioxidants. This shows the dichotomous behaviour of phytocompounds. Presence of copper enhances the damage in DNA obtained from blood sample of diabetic subjects. Spectral absorbance studies display increase in absorbance with increasing DNA concentration on binding to caffeine, naringenin and hypochromism on binding to curcumin. We speculate that unwinding of DNA makes it vulnerable for attack by the metal-aided free radicals and intercalation offers protection against attack. To conclude, nutraceuticals like caffeine, curcumin, and naringenin may act as inducers as well as quenchers of oxidative free radicals. This dichotomous behaviour can be applied wisely to combat oxidative stress arising from diabetes and cancer.

## Figures and Tables

**Table 1:** Clinical characteristics of studied subjects.

Subjects	Postprandial blood glucose level (mg/dL)	Age (Year)	Smoking habits	Other symptoms
Patient 1	452	38	Chain smoker	Delayed wound healing, boils, high blood pressure (140/100)
Patient 2	379	55	Nonsmoker	Knee pain, poor eyesight
Patient 3	337	59	Nonsmoker	Knee and back pain, numbness of feet
Patient 4	308	51	Nonsmoker	None
Patient 5	411	50	Nonsmoker	Poor eyesight, high blood pressure (150/90)
Patient 6	427	50	Nonsmoker	Poor eye sight, boils

All the patients were newly diagnosed with Type 2 Diabetes Mellitus. Therefore, none of the patients were on any drug administration and insulin treatment.

## References

[1] Cooke MS, Evans MD, Dizdaroglu M, Lunec J (2003). Oxidative DNA damage: mechanisms, mutation, and disease. *The FASEB Journal*.

[2] Ebara M, Fukuda H, Hatano R (2000). Relationship between copper, zinc and metallothionein in hepatocellular carcinoma and its surrounding liver parenchyma. *Journal of Hepatology*.

[3] Gunther MR, Hanna PM, Mason RP, Cohen MS (1995). Hydroxyl radical formation from cuprous ion and hydrogen peroxide: a spin-trapping study. *Archives of Biochemistry and Biophysics*.

[4] Hyslop PA, Hinshaw DB, Halsey WA (1988). Mechanisms of oxidant-mediated cell injury. The glycolytic and mitochondrial pathways of ADP phosphorylation are major intracellular targets inactivated by hydrogen peroxide. *The Journal of Biological Chemistry*.

[5] Halliwell B (1996). Antioxidants in human health and disease. *Annual Review of Nutrition*.

[6] Bloch-Damti A, Potashnik R, Gual P (2006). Differential effects of IRS1 phosphorylated on Ser307 or Ser632 in the induction of insulin resistance by oxidative stress. *Diabetologia*.

[7] Perron NR, Hodges JN, Jenkins M, Brumaghim JL (2008). Predicting how polyphenol antioxidants prevent DNA damage by binding to iron. *Inorganic Chemistry*.

[8] Shi X, Dalal NS, Jain AC (1991). Antioxidant behaviour of caffeine: efficient scavenging of hydroxyl radicals. *Food and Chemical Toxicology*.

[9] Svilaas A, Sakhi AK, Andersen LF (2004). Intakes of antioxidants in coffee, wine, and vegetables are correlated with plasma carotenoids in humans. *The Journal of Nutrition*.

[10] Keijzers GB, de Galan BE, Tack CJ, Smits P (2002). Caffeine can decrease insulin sensitivity in humans. *Diabetes Care*.

[11] Reunanen A, Heliovaara M, Aho K (2003). Coffee consumption and risk of type 2 diabetes mellitus. *The Lancet*.

[12] van Dam RM, Feskens EJM (2002). Coffee consumption and risk of type 2 diabetes mellitus. *The Lancet*.

[13] van Dam RM, Hu FB (2005). Coffee consumption and risk of type 2 diabetes: a systematic review. *Journal of the American Medical Association*.

[14] Wedick NM, Brennan AM, Sun Q, Hu FB, Mantzoros CS, van Dam RM (2011). Effects of caffeinated and decaffeinated coffee on biological risk factors for type 2 diabetes: a randomized controlled trial. *Nutrition Journal*.

[15] Jiang X, Zhang D, Jiang W (2014). Coffee and caffeine intake and incidence of type 2 diabetes mellitus: a meta-analysis of prospective studies. *European Journal of Nutrition*.

[16] Greer F, Hudson R, Ross R, Graham T (2001). Caffeine ingestion decreases glucose disposal during a hyperinsulinemic-euglycemic clamp in sedentary humans. *Diabetes*.

[17] Ohnaka K, Ikeda M, Maki T (2012). Effects of 16-week consumption of caffeinated and decaffeinated instant coffee on glucose metabolism in a randomized controlled trial. *Journal of Nutrition and Metabolism*.

[18] Singh U, Barik A, Singh BG, Priyadarsini KI (2011). Reactions of reactive oxygen species (ROS) with curcumin analogues: structure-activity relationship. *Free Radical Research*.

[19] Srinivasan M (1972). Effect of curcumin on blood sugar as seen in a diabetic subject. *Indian Journal of Medical Sciences*.

[20] Usharani P, Mateen AA, Naidu MUR, Raju YSN, Chandra N (2008). Effect of NCB-02, atorvastatin and placebo on endothelial function, oxidative stress and inflammatory markers in patients with type 2 diabetes mellitus: a randomized, parallel-group, placebo-controlled, 8-week study. *Drugs in R & D*.

[21] Kumar A, Bora U (2013). Interactions of curcumin and its derivatives with nucleic acids and their implications. *Mini-Reviews in Medicinal Chemistry*.

[22] Felgines C, Texier O, Morand C (2000). Bioavailability of the flavanone naringenin and its glycosides in rats. *American Journal of Physiology—Gastrointestinal and Liver Physiology*.

[23] Erlund I, Meririnne E, Alfthan G, Aro A (2001). Plasma kinetics and urinary excretion of the flavanones naringenin and hesperetin in humans after ingestion of orange juice and grapefruit juice. *The Journal of Nutrition*.

[24] Bhattacharya S, Oksbjerg N, Young JF, Jeppesen PB (2013). Caffeic acid, naringenin and quercetin enhance glucose stimulated insulin secretion and glucose sensitivity in INS-1E cells. *Diabetes, Obesity and Metabolism*.

[25] Annadurai T, Thomas PA, Geraldine P (2013). Ameliorative effect of naringenin on hyperglycemia-mediated inflammation in hepatic and pancreatic tissues of Wistar rats with streptozotocin-nicotinamide-induced experimental diabetes mellitus. *Free Radical Research*.

[26] Meneghini R (1997). Iron homeostasis, oxidative stress, and DNA damage. *Free Radical Biology & Medicine*.

[27] Fiorentino TV, Prioletta A, Zuo P, Folli F (2013). Hyperglycemia-induced oxidative stress and its role in diabetes mellitus related cardiovascular diseases. *Current Pharmaceutical Design*.

[28] Stoner GD, Wang L-S, Casto BC (2008). Laboratory and clinical studies of cancer chemoprevention by antioxidants in berries. *Carcinogenesis*.

[29] Schweigert N, Zehnder AJB, Eggen RIL (2001). Chemical properties of catechols and their molecular modes of toxic action in cells, from microorganisms to mammals. *Environmental Microbiology*.

[30] Zheng L-F, Wei Q-Y, Cai Y-J (2006). DNA damage induced by resveratrol and its synthetic analogues in the presence of Cu (II) ions: mechanism and structure-activity relationship. *Free Radical Biology & Medicine*.

[31] Samuni AM, Chuang EY, Krishna MC (2003). Semiquinone radical intermediate in catecholic estrogen-mediated cytotoxicity and mutagenesis: chemoprevention strategies with antioxidants. *Proceedings of the National Academy of Sciences of the United States of America*.

[32] Azmi AS, Bhat SH, Hadi SM (2005). Resveratrol-Cu(II) induced DNA breakage in human peripheral lymphocytes: implications for anticancer properties. *The FEBS Letters*.

[33] Malik A, Azam S, Hadi N, Hadi SM (2003). DNA degradation by water extract of green tea in the presence of copper ions: implications for anticancer properties. *Phytotherapy Research*.

[34] Li Z, Yang X, Dong S, Li X (2012). DNa breakage induced by piceatannol and copper(II): mechanism and anticancer properties. *Oncology Letters*.

[35] Leung MH, Pham DT, Lincoln SF, Kee TW (2012). Femtosecond transient absorption spectroscopy of copper(II)-curcumin complexes. *Physical Chemistry Chemical Physics*.

[36] Barik A, Mishra B, Kunwar A (2007). Comparative study of copper(II)-curcumin complexes as superoxide dismutase mimics and free radical scavengers. *European Journal of Medicinal Chemistry*.

[37] Basu A, Kumar GS (2013). Biophysical studies on curcumin-deoxyribonucleic acid interaction: spectroscopic and calorimetric approach. *International Journal of Biological Macromolecules*.

[38] Hegde AH, Prashanth SN, Seetharamappa J (2012). Interaction of antioxidant flavonoids with calf thymus DNA analyzed by spectroscopic and electrochemical methods. *Journal of Pharmaceutical and Biomedical Analysis*.

[39] Mello LD, Pereira RMS, Sawaya ACHF, Eberlin MN, Kubota LT (2007). Electrochemical and spectroscopic characterization of the interaction between DNA and Cu(II)-naringin complex. *Journal of Pharmaceutical and Biomedical Analysis*.

